# Comparable effects on tear film parameters after femtosecond laser-assisted and conventional cataract surgery

**DOI:** 10.1007/s10792-020-01532-z

**Published:** 2020-08-03

**Authors:** Marc Schargus, Svetlana Ivanova, Gesa Stute, H. Burkhard Dick, Stephanie C. Joachim

**Affiliations:** 1 Department of Ophthalmology, Heinrich-Heine-University Düsseldorf, Moorenstraße 5, 40225 Düsseldorf, Germany; 2 Asklepios Nord Eye Hospital, Hamburg, Germany; 3 Institute for Vision Science, University Eye Clinic, Ruhr-University Bochum, Bochum, Germany; 4 Experimental Eye Research Institute, University Eye Clinic, Ruhr-University Bochum, Bochum, Germany

**Keywords:** Dry eye, MMP-9, Tear film parameters, Femtosecond laser-assisted cataract surgery, Conventional cataract surgery

## Abstract

**Purpose:**

Dry eye symptoms after conventional cataract surgery are a very common problem. Until now, only few data are available on objective tear film parameters in regard to femtosecond laser-assisted cataract surgery (LCS). Aim of this study was therefore to analyze and compare tear film parameter changes between LCS and conventional cataract surgery.

**Methods:**

A consecutive group of 34 patients, scheduled for cataract surgery, were randomly selected for either LCS or conventional cataract surgery (17 patients/group). Tear film assessments including tear film osmolarity, Schirmer test, MMP-9 analysis via quantitative ELISA, corneal sensitivity, corneal fluorescein staining, and conjunctival fluorescein staining were sequentially evaluated pre- as well as 1 and 3 months postoperatively.

**Results:**

Both groups showed no significant difference in baseline characteristics. All surgeries were performed without any complications. After 1 and 3 months, there was no statistically significant difference in regard to tear film osmolarity (1 month: *p* = 0.81, 3 months: *p* = 1.0), Schirmer test (1 month: *p* = 0.35, 3 month: *p* = 0.08), and MMP-9 concentration (1 month: *p* = 0.36, 3 month: *p* = 0.28) between the two groups.

**Conclusions:**

Neither LCS nor conventional cataract surgery affected objective tear film parameters significantly during our 3-month postoperative observation period. Hence, both surgical techniques can be equally used to treat patients without prior dry eye symptoms.

## Introduction

Cataract surgery has evolved rapidly over the past decades, and new technologies, like femtosecond laser-assisted cataract surgery (LCS), were adopted to enhance optical and anatomical results and safety of this surgical procedure [1]. Dry eye disease (DED) is a common condition in elderly people. Numerous reports were published on tear film alterations after cataract surgery in the past, dealing with subjective symptoms of DED and ocular discomfort within the first 1–3 months after surgery [2–4]. Several evaluation techniques exist to diagnose DED. Common tests include patient symptom questionnaires, tear film breakup time (TBUT), Schirmer test, fluorescein as well as lissamine green staining and meibomian secretion scoring [5]. However, most of these tests lack objectivity and specificity or are prone to user-dependent analytical errors [6]. Tear film osmolarity (TFO) and matrix metalloproteinases (MMP) level analysis promise to be more reliable DED tests than subjective scorings or tests. High TFO values can affect the stability of the tear film by modifying the interaction between tear film lipids and proteins, damaging epithelial cell membranes, triggering inflammation, and stimulating corneal nerves [5, 7]. Several studies came to the conclusion that TFO correlates well with DED severity [8–10]. Giannaccare et al. [11] exclusively investigated objective tear film parameters in a cross-sectional study using various automated examination methods but without testing MMP-9. They concluded that an automated noninvasive examination of the ocular surface with several examination parameters can be a useful screening tool for dry eyes and meibomian gland dysfunction where individual tests are not sufficiently meaningful. MMPs are produced in ocular surface diseases and are important contributors to corneal destruction and perforation [12]. Numerous studies noted higher MMP-9 tear levels in patients with severe disorders affecting the ocular surface, like Graft versus Host disease, Sjögren syndrome, or after corneal surgeries, including Laser in situ Keratomileusis [13].

To our best knowledge, until now, there are only two publications assessing tear film parameters after LCS and conventional cataract surgery, but without assessing the objective tear film parameters TFO and MMP-9 [14, 15]. The aim of this study was therefore to determine prospectively whether there is a significant difference in objective tear film parameters before and after LCS and conventional cataract surgery during a three-month postoperative period.

## Methods

### Subjects

This study received approval of the ethics committee of the Ruhr University in Bochum, Germany, and conformed to the tenets of the Declaration of Helsinki. The study was registered in the German Register of Clinical Studies (DRKS; registration number: DRKS00021773).

Patients were enrolled in this prospective, randomized, single-center study between March 2014 and March 2015. They had a visually significant cataract (NC2 to NC5 regarding LOCS III classification [16]) and corrected distance visual acuity decreased by minimum to 0.1 log MAR in both eyes, a dilated pupil width of 6.0 mm or greater, and were willing to volunteer for the trial after giving written consent. Cataract surgery was performed only on one eye during the study period. The exclusion criteria included corneal scars, corneal diseases, glaucoma, pseudoexfoliation syndrome, zonular weakness, history of ocular surgery, active or past inflammations, reduced compliance, age younger than 50 years, any usage of eyedrops within 1 month prior to surgery, or participation in another clinical study within 30 days of the preoperative visit.

### Dry eye examinations

Dry eye examinations included a variety of tests, which were done in the following sequence: tear film osmolarity measurement, Schirmer test (these samples were later used for MMP-9 analysis), corneal sensitivity, corneal fluorescein staining, and conjunctival lissamine green staining. The preoperative examination was carried out 1 week before surgery and the follow-up examinations 1 and 3 months after surgery.

Tear film osmolarity was determined with the TearLab Osmolarity System (TearLab Corp., San Diego, CA, USA) using a 50 nL sample of tear film obtained from the lateral canthus of the tear meniscus. Measurements were done at similar daytime between 9:00 and 12:00 in the morning within a closed room with similar temperature and humidity to avoid any influence on the measurements. Osmolarity values were measured in mOsm/L.

A 5-minute Schirmer test was performed with sterile strips without anesthetic inserted at the border of the medial to the lateral third of the lower lid margin with the lids closed. After completing the test, the strips were immediately frozen at − 80 °C for further MMP-9 analysis.

For baseline DED diagnostics, three more DED-related tests were obtained preoperatively (corneal esthesiometry, corneal staining, and conjunctival staining). Corneal sensation was measured in all subjects in the central area of the cornea with the Cochet–Bonnet [17] esthesiometer (Luneau Ophtalmologie, Chartres, France). Mechanical stimulation was performed by touching the corneal surface with the tip of a retractable, flexible monofilament nylon thread, 60 mm long and 0.12 mm in diameter. As the flexible nylon thread was shortened, the stiffness increased. Corneal tactile sensation was expressed as the maximum length of filament that generated a tactile sensation. For corneal staining, 5 μL of a 2% sodium fluorescein solution was instilled using a micropipette and corneal staining was evaluated under cobalt blue illumination 2.5–3.0 min after fluorescein instillation. While conjunctival staining was assessed 2.5–3.0 min after 10 μL of a 1% sodium lissamine green dye was instilled. Corneal and conjunctival staining levels were graded according to the NEI/Industry Workshop scale [18].

Full examination of anterior and posterior eye segment was done by slit lamp and biomicroscopy including lens opacity evaluation using the LOCS III classification with dilated pupil [16]. Corrected distance visual acuity (CDVA) was measured preoperatively and after 1 and 3 months using ETDRS charts.

### Cataract surgeries

Three days before surgery, all patients were treated with topical ofloxacin four times daily. No nonsteroidal anti-inflammatory drugs were administered.

Randomization was done by numbered envelopes, which were opened after inclusion to the study. The randomization scheme was generated by using the Web site Randomization.com (http://www.randomization.com). Then, surgery was performed with either the laser system (CATALYS^®^ Precision Laser System; Johnson and Johnson, NJ, USA) or a Stellaris phacoemulsification device (Bausch & Lomb, Rochester, NY, USA). All surgical procedures were carried out by a single experienced surgeon (HBD) at the Department of Ophthalmology, Ruhr-University Bochum, Germany. At the 3- and 9-o’clock positions, two 1.2-mm clear corneal side incisions with a self-sealing Blumenthal lance and on the steep corneal axis a 2.75-mm two-step clear cornea incision were created. Other surgical steps were performed as described before [19].

Topical ofloxacin eyedrops (floxal 3 mg/mL, Bausch & Lomb - Dr. Mann Pharma, Germany) were administered three times daily for 5 days in both patient groups. Additionally, dexamethasone eyedrops (dexasine 1 mg/mL, Alcon, USA) were administered four times daily for the first week, after which the dosage was gradually tapered over 4 weeks.

### MMP-9 ELISA analysis

The top 10 mm of all Schirmer strips were cut off and eluted with 500 μL phosphate-buffered saline plus 1% Triton X-100 over night. This way tear samples were obtained for subsequent MMP-9 analysis [20]. A colorimetric solid-phase sandwich human MMP-9 ELISA Kit (Affymetrix eBioscience, Wien, Austria) was used to determine MMP-9 concentrations in human tear fluid. Ten microliters per sample was used, and the assay was performed according to the manufacturer’s instructions. Briefly, assay buffer and samples were added to wells, before the biotin conjugate was added, and plates were placed on a shaker at room temperature for 2 h. After several washing steps, wells were incubated with streptavidin-HRP at room temperature for 1 h, followed by washing steps. Then, the TMB substrate solution was added for 10 min followed by stop solution. The measurements of the standards and all samples were performed at a wavelength of 405 nm with a microplate reader (AESKU.Reader with Gen5 ELISA Software; AESKU.DIAGNOSTICS, Wendelsheim, Germany) [10].

### Statistics

Main outcome measurements were the TFO, MMP-9, and Schirmer test values before surgery as well as 1 and 3 months postoperatively in both groups. Secondary outcome measurements included evaluation of best corrected visual acuity and complications due to LCS. All descriptive statistical analysis was performed using Statistica software (version 13; Dell, Tulsa, Ok, USA). *T* test was used to test for differences in both groups at each time point. Graphs display mean values ± SEM ± SD and tables mean values ± SD unless stated otherwise. *p* values below 0.05 were considered statistically significant.

## Results

Thirty-four eyes of 34 patients (18 female, 16 male) were included and analyzed within the two groups with a follow-up period of 3 months. Baseline characteristics are shown in Table 1. The LCS group included 17 patients (eight female, nine male) with an average age of 67.4 ± 9.7 years. The conventional group included 17 patients (ten female, seven male) with an average age of 66.0 ± 7.5 years.

**Table 1:** Baseline characteristics of both groups, conventional cataract surgery and femtosecond laser-assisted cataract surgery (LCS)

Group	Conventional cataract surgery	LCS
Number of patients	17	17
Mean age ± standard deviation (years)	66.0 ± 7.5	67.4 ± 9.7
Sex (male/female)	7/10	9/8
Eye (right/left)	7/10	10/7
Mean cataract stage (LOCS level)	3.6 ± 1.1	3.8 ± 1.4
Corneal sensitivity (mm/S)	5.57 ± 0.17	5.14 ± 0.39
Corneal fluorescein staining (points)	0.06 ± 0.14	0.010 ± 0.11
Conjunctival lissamine green staining (points)	0.44 ± 0.19	0.28 ± 0.22

Preoperative LOCS III grading showed similar distribution of the average grading in the LCS (3.6 ± 1.1) and conventional group (3.8 ± 1.4; *p* = 0.69). Mean preoperative CDVA was 0.39 ± 0.18 log MAR in the LCS compared to 0.38 ± 0.17 log MAR in the conventional group. Mean CDVA in both groups showed significant improvement 3 months after surgery to 0.87 ± 0.13 log MAR in LCS and 0.77 ± 0.22 in the conventional group. There was no statistically significant difference between the two groups (*p* = 0.11).

Preoperative TFO revealed comparable values in both groups (LCS: 301.9 ± 14.4 mOsml/L; conventional group: 299.8 ± 12.9 mOsml/L; *p* = 0.65). Preoperatively, the mean Schirmer test values were also not significantly different between groups (LCS: 13.5 ± 7.9 mm; conventional group: 12.7 ± 8.2 mm; *p* = 0.78). Mean MMP-9 values also showed no significant difference between the two groups (LCS: 18.4 ± 26.2 ng/mL, conventional group: 12.3 ± 21.1 ng/mL; *p* = 0.49). In addition, other more subjective tests to evaluate the DED stage did not display any significant differences. Corneal sensitivity (LCS: 0.010 ± 0.11; conventional: 0.06 ± 0.14), corneal fluorescein staining (LCS: 5.14 ± 0.39; conventional: 5.57 ± 0.17), and conjunctival lissamine green staining (LCS: 0.28 ± 0.22; conventional: 0.44 ± 0.19) were comparable within the groups.

**Fig. 1:**
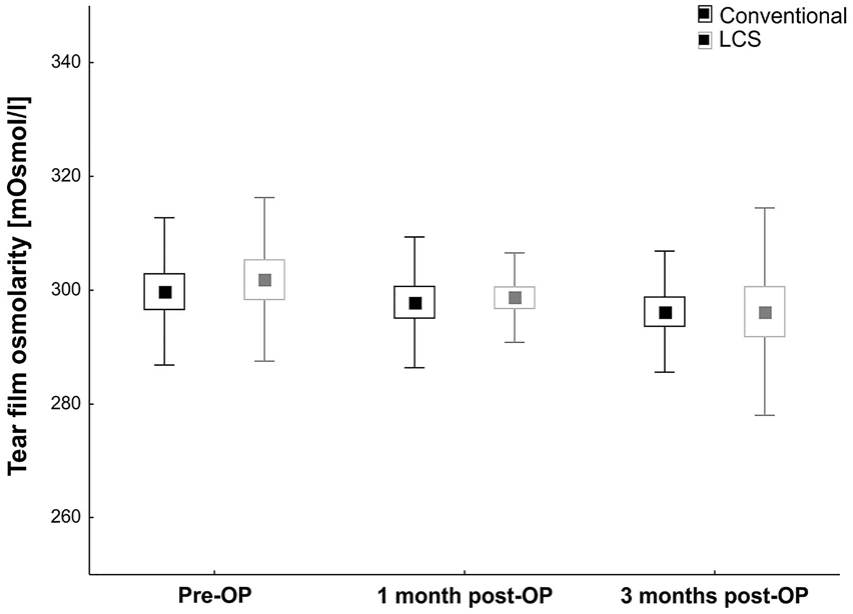
Mean TFO values preoperatively as well as 1 and 3 months postoperatively in both groups. Black bars show the conventional cataract surgery group, and gray bars show LCS group. Values are mean ± SEM ± SD

The results after 1 and 3 months showed no significant difference between the groups regarding TFO (1 month: LCS: 298.7 ± 11.5 mOsml/L vs. conventional: 297.9 ± 11.5 mOsml/L; *p* = 0.81; 3 months: LCS: 296.2 ± 18.2 mOsml/L vs. conventional: 296.2 ± 10.7 mOsml/L; *p* = 1.0; Table 2; Fig. 1).

**Table 2:** MMM-9, Schirmer test, and tear osmolarity values in both groups before surgery (per-OP) as well as 1 and 3 months after surgery

	Pre-op			1 month post-op			3 months post-op		
	Convent. cataract surgery	LCS	*p*-value	Convent. cataract surgery	LCS	*p*-value	Convent. cataract surgery	LCS	*p*-value
MMP-9 (ng/mL)	12.3 ± 21.1 0.1–24.5	18.4 ± 26.2 4.5–32.4	0.49	14.1 ± 20.9 2.9–25.1	8.9 ± 6.2 5.6–12.2	0.36	8.5 ± 8.6	12.1 ± 9.8 6.8–17.3	0.28
Schirmer test (mm)	12.7 ± 8.2 8.5–16.9	13.5 ± 7.9 9.4–17.6	0.78	14.9 ± 8.2 10.7–19.2	12.3 ± 7.9 8.2–16.4	0.35	17.2 ± 8.7 12.8–21.7	12.0 ± 8.3 7.7–16.3	0.08
Tear osmolarity (mOsml/L)	299.8 ± 12.9 293.1–306.4	301.9 ± 14.4 294.5–309.2	0.65	297.9 ± 11.5	298.7 ± 11.5 294.7–302.8	0.81	296.2 ± 10.7 290.7–301.7	296.2 ± 18.2 286.9–305.6	1.0

All data are displayed as mean ± SD plus 95% confidence interval. Both groups at each point in time were compared via *t* test

**Fig. 2:**
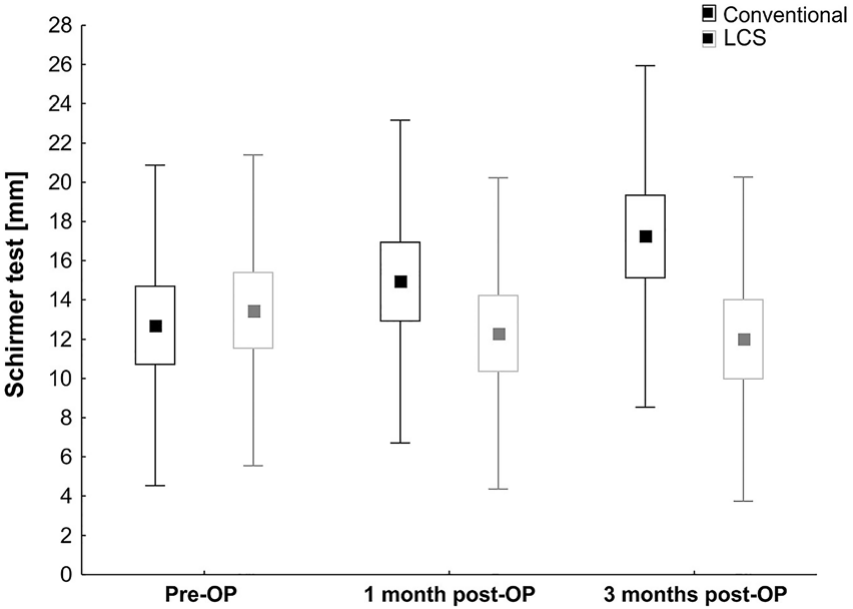
Bar graph showing Schirmer values preoperatively and also 1 and 3 months postoperatively in both groups. Conventional cataract surgery data are displayed in black and LCS data in gray. Values are mean ± SEM ± SD

The same observation was made in terms of the Schirmer test, at 1 month (LCS: 12.3 ± 7.9 mm vs. conventional: 14.9 ± 8.2 mm, *p* = 0.35) and 3 months (LCS: 12.0 ± 8.3 mm vs. conventional: 17.2 ± 8.7 mm, *p* = 0.08; Table 2; Fig. 2); comparable values were recorded in both groups.

**Fig. 3:**
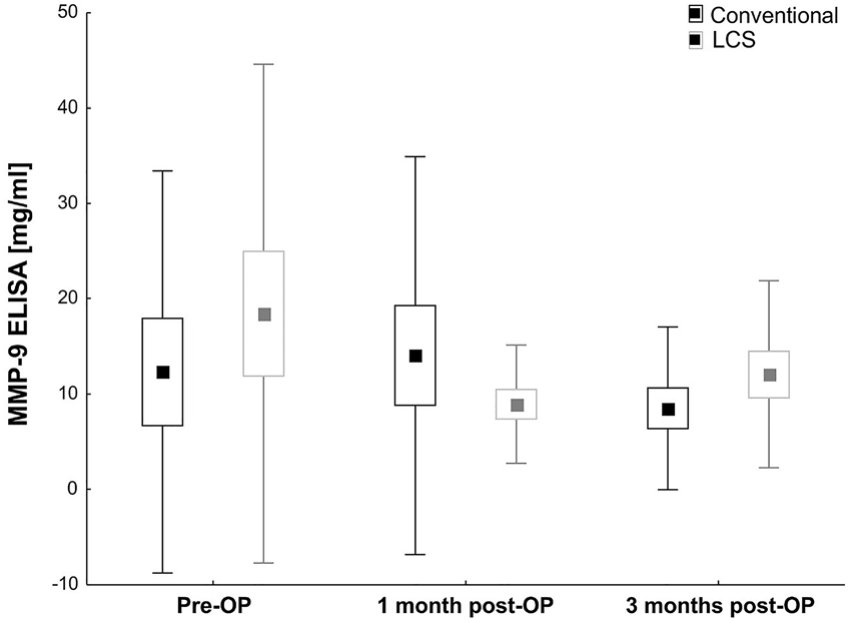
Mean MMP-9 values obtained via ELISA from both groups are displayed. MMP-9 values were measured preoperatively and 1 and 3 months after surgery. Black bars show conventional cataract surgery values, and gray bars show LCS values. Values are mean ± SEM ± SD

MMP-9 measurements at 1 month (LCS: 8.9 ± 6.2 ng/mL vs. conventional: 14.1 ± 20.9 ng/mL; *p* = 0.36) and 3 months (LCS: 12.1 ± 9.8 ng/mL vs. conventional: 8.5 ± 8.6 ng/mL; *p* = 0.28) were also very similar in the two groups (Table 2; Fig. 3).

No patient in the LCS or conventional group showed complications intraoperatively or due to the surgical procedure within the follow-up time of 3 months.

## Discussion

Cataract surgery is the most common surgery worldwide, and DED is considered a common ocular condition affecting 5–35% of the worldwide population [21]. After cataract surgery, patients often complain of DED symptoms, which can be a significant cause of vision impairment and reducing patients’ quality of life [3, 14, 22, 23]. Known factors for postoperative DED symptoms were evaluated over decades, including preoperative subclinical dry eye, disinfection with iodine [3], expression of inflammatory cytokines [24], prolonged light exposure during surgery, total length of surgery [2], usage of different lid speculums, and the size of corneal incisions [2]. Due to optimized and modern techniques, short surgical time, and individual optimized pre- and postoperative treatments, rates of ocular discomfort and visual disturbance could likely be reduced to a minimum postoperatively [25]. But still, some patients suffer from dry eye symptoms. Hence, aim of this study was to analyze objective markers for dry eye before and after cataract surgery using two surgical techniques.

The introduction of the femtosecond laser into the field of cataract surgery offers a new technology in several steps of the surgical procedure. Different laser systems from different vendors have been established in LCS through the last years using different docking systems and laser parameters. A metanalysis of more than 6.000 eyes treated by LCS showed no significant difference in CDVA and mean absolute error [26]. However, to date, just two studies examined the differences in dry eye-related postsurgical differences between LCS and conventional cataract surgery [14, 15]. A prospective consecutive nonrandomized comparative cohort study by Yu et al. [14] noted in 137 patients that underwent LCS or conventional cataract surgery DED symptoms postoperatively, regardless of the surgical technique. While this study examined several DED symptoms, unfortunately there was only a follow-up period of 1 month after surgery and they did not examine MMP-9 or TFO, which are well-recognized objective pathogenetic markers for DED [10]. Additionally, they used a different laser system (LensX, Alcon Inc., TX, USA) than in our study and a different postoperative topical treatment scheme (topical dexamethasone four times per day, tobramycin for 2 weeks, and pranoprofen for 1 month). Their study showed a significantly higher number of DED patients postoperatively (> 50%) with a peak at 1 week postoperatively. Schirmer test values did not show significant difference between groups after 1 month. The results from this study confirm our findings, and we also noted no significant difference in Schirmer test values after 1 and 3 months.

A recently published study from Shao et al. [15] examined some dry eye parameters in a group of 150 eyes with LCS and 150 eyes with conventional cataract surgery. Here, authors also examined few nonobjective DED with a follow-up of 3 months. While they noted a worsening of DED symptoms via OSDI and corneal fluorescein staining until week 1 after surgery, there were no statistical significant differences in OSDI, corneal fluorescein staining, breakup time, and tear meniscus height after 3 months. This study also used another laser system (LensX, Alcon Inc., TX, USA) and a different postoperative treatment scheme [combination of topical dexamethasone/antibiotics (tobramycin)/nonsteroidal anti-inflammatory drug (pranoprofen) four times per day for 1 week and then decreased by one eyedrop/day every week].

We observed no significant difference between LCS and conventional cataract surgery in any of the applied objective tear film parameters (TFO, MMP-9, and Schirmer test) after 1 and 3 months postoperatively. There can be different explanations for our study results, which are in contrast to previously published data from other studies. Gupta et al. [27] examined the prevalence of DED symptoms in patients presenting for cataract surgery. They noted that 54% of the patients had osmolarity readings > 307 mOsmol/L and 65% had abnormal MMP-9 levels (> 40 ng/mL), whereas 72% reported no or just mild DED symptoms (OSDI < 22 points). Therefore, many study results on DED after cataract surgery may be triggered by previously not detected subclinical DED [26]. Our preoperative objective examinations displayed a typical cross section of patients without significant pathological values in TFO, MMP-9, or Schirmer test, so extreme outliners were not present in our group of patients. This might be an explanation of stable postoperative examination values without worsening of DED signs in this study.

Surgery setting in our study was optimal for LCS as well as for conventional cataract surgery with a very experienced surgeon performing both procedures without changing the working place between LCS procedure and the following manual part of the surgery in an overall of 6–8 min complete surgical time. Exposition of the ocular surface to different influences like air, lid speculum, or eyedrops therefore was only minimally different from the conventional cataract surgery group.

Mechanical irritation to the conjunctiva causes damage to epithelial and goblet cells and can be a reason for tear film changes [24]. Therefore, the impact of the suction ring in LCS to the peri-limbic conjunctiva cannot be determined exactly. Yu et al. and Shao et al. [14, 15] showed a complete regression of corneal fluorescein staining from baseline to month 1. As it is known, suction rings can cause a decrease of conjunctival goblet cells after LASIK as they are used also in the LensX LCS system. The CATALYS^®^ Precision Laser System uses a Liquid Optics interface to minimize conjunctiva contact. Lissamine green staining would be helpful in future trials to determinate the impact of suction rings, while it provides information on the damage of the epithelial conjunctival tissue.

Transection of corneal innervations due to cutting nerve fibers from the corneal subbasal nerve plexus during laser in situ keratomileusis or corneal incisions due to cataract surgery does lower the corneal sensitivity as described in previous studies [24, 28]. But most of these studies rely on older techniques, with greater corneal incision size, which are no longer state of the art in modern cataract surgery. In our study, only a small incision size was performed, as stated in the Methods section. Furthermore, all studies in this field used a different pre- and postoperative topical treatment. We administered topical steroids for 4 weeks, while several other studies just use NSAIDs on different regimes or topical combinations of steroids and antibiotics; hence, this additionally triggers DED symptoms [14].

A limitation of our study might be the small sample size of 34 patients. The portfolio of dry eye tests is large and could be expanded in most studies, also in ours. We focused our tests on the main objective test values for follow-up examinations. Standardized symptom questionnaires, like OSDI, are not useful for these trials, since several questions target visual function, which changes due to cataract surgery. New questionnaires must be developed to target this question. Other subjective tests were excluded for follow-up. An automated objective measurement of TBUT would also have been desirable, but a corresponding test option was unfortunately not available. Due to the fact that corneal fluorescein staining and conjunctival lissamine green staining were normal, we suspect that TBUT was not in the pathological range either.

In conclusion, our results suggest that patients without abnormal tear film settings before cataract surgery, using modern techniques like LCS and conventional cataract surgery, show no significant changes in TFO, MMP-9 levels, and Schirmer test after 1 and 3 months. By continuous development of less invasive cataract surgical procedures, the rate of postoperatively dry eye symptoms will further decrease.
